# Supplementary material for: Content-rich biological network constructed by mining PubMed abstracts
Source: BMC Bioinformatics. 2004 Oct 8;5:147. doi: 10.1186/1471-2105-5-147 (PMC528731; doi:10.1186/1471-2105-5-147)
Supplement: Additional File 2 — The original results of the above study (non-essential files are deleted to keep the file size under the limit set by BMC bioinformatics). [file 1471-2105-5-147-S2.bz2 › chilibotAdditionalFile2/dip20/572ID10722747E10939/html/NTRK1_BDNF.html]

 


 **NTRK1** and **BDNF** 
  
Found 566 abstracts in PubMed, retrieved 20.  
 

 What does Google say? 
 PDF only 
| .edu only 

---

**Interactive relationship** (e.g. stimulation, inhibition, etc)

**Inhibitory relationship**- Moreover, the neuroprotective effect of  **BDNF**  and LIGA20 was abolished in neurons exposed to the Trk  [ **NTRK1** ]  tyrosine kinase inhibitor k252a, demonstrating a relationship between neuroprotection and activation of Trk  [ **NTRK1** ]  receptors.  Ref: 12829403 Neurotox Res, 2002
**Neutral relationship**- Trk  [ **NTRK1** ]  receptors are a family of three receptor tyrosine kinases, each of which can be activated by one or more of four neurotrophins nerve growth factor, brain derived neurotrophic factor  [ **BDNF** ] , and neurotrophins 3 and 4.  Ref: 12676795 Annu Rev Biochem< MedlineTA>Annu Rev Biochem, 2002

**Non-interactive relationship** (e.g. studied together, co-existance, homology, etc.)

- To test this, we infused the cortex with an inhibitor of Trk  [ **NTRK1** ]  receptor tyrosine kinases, anti TrkB IgG1, anti  **BDNF** , and anti neurotrophin 4 5 antibodies.  Ref: 12736347 J Neurosci, 2003
- OBJECTIVE There are preclinical data showing that fluoxetine stimulated expression of Brain Derived Neurotrophic Factor  [ **BDNF** ]   **BDNF**  and its specific tyrosine kinase receptor  [ **NTRK1** ] , and caused neuritic elongation and increased dendritic branching density of CA3 hippocampal pyramidal cell neurons in rodents.  Ref: 12779183 Int J Psychiatry Med, 2002
- Both flat and process bearing ECs were immunopositive for brain derived neurotrophic factor  [ **BDNF** ] , nerve growth factor, neurotrophin 4, and TrkB but not TrkA  [ **NTRK1** ] .  Ref: 12555206 Glia, 2003
- **BDNF**  s ability to rescue the cells from cisplatin induced cell death was inhibited by treatment with the Trk  [ **NTRK1** ]  tyrosine kinase inhibitor, K252a, and the phosphatidylinositol 3 kinase 3 kinase inhibitor, LY294002.  Ref: 12691830 Cancer Lett, 2003
- We found that 1 trk  [ **NTRK1** ]  C, a receptor for neurotrophin 3 NT 3, is dominantly expressed in RCEC, but trk  [ **NTRK1** ]  B, a receptor for brain derived neurotrophic factor  [ **BDNF** ] , is dominantly expressed in RAEC.  Ref: 12745089 Biochem Biophys Res Commun, 2003
- Western blot analysis confirmed these results, showing that the lacrimal gland expresses NGF,  **BDNF** , NT3, and NT4 as well as the NTrs TrkA  [ **NTRK1** ] , TrkB, and TrkC and the p75 protein.  Ref: 12882781 Invest Ophthalmol Vis Sci, 2003
- In contrast, null mutant mice for TrkB, the tyrosine kinase receptor  [ **NTRK1** ]  for brain derived neurotrophic factor  [ **BDNF** ]  and neurotrophin 4, displayed a clear loss of locus coeruleus noradrenergic neurons.  Ref: 12810600 Development, 2003
- RT PCR showed positive bands at the appropriate sizes for NGF,  **BDNF** , NT3, and NT4, and for the receptors TrkA  [ **NTRK1** ] , TrkB, TrkC, and p75.  Ref: 12882781 Invest Ophthalmol Vis Sci, 2003
- To determine which of the neurotrophins NTs nerve growth factor NGF, brain derived neurotrophic factor  [ **BDNF** ]   **BDNF** , neurotrophin 3 NT3, and neurotrophin 4 5 NT4 and their receptors NTrs, TrkA  [ **NTRK1** ] , TrkB, TrkC, and p75, are present in the adult rat lacrimal gland.  Ref: 12882781 Invest Ophthalmol Vis Sci, 2003
- Cultured cortical progenitors express  **BDNF**  and NT 3, as well as their receptors TrkB tyrosine kinase receptor  [ **NTRK1** ]  B and TrkC.  Ref: 12832539 J Neurosci, 2003
- Lithium neuroprotection against excitotoxicity is inhibited by a  **BDNF**  neutralizing antibody and K252a, a Trk  [ **NTRK1** ]  antagonist.  Ref: 12701214 Seishin Shinkeigaku Zasshi, 2003
- Consistent with the presence of trkB but not trkA  [ **NTRK1** ] ,  **BDNF**  causes rapid phosphorylation of MAP kinases ERK1 and ERK2, but NGF does not.  Ref: 12672017 J Neurobiol, 2003
- In this work the expression and anatomical localization of NGF,  **BDNF**  and NT 3 and of corresponding high affinity receptors TrkA  [ **NTRK1** ] , TrkB full length and truncated TR isoforms, TrkC and of the low affinity p75 receptor were assessed in surgical samples from adult human lung by reverse transcriptase polymerase chain reaction RT PCR, Western blot and immunohistochemistry.  Ref: 12791675 Am J Respir Cell Mol Biol< MedlineTA>Am J Respir Cell Mol Biol, 2003
- Moreover, trophic effects of OLG conditioned medium were partially blocked by K252a, a trk  [ **NTRK1** ]  tyrosine kinase inhibitor, and by neutralizing anti  **BDNF**  or anti NT 3 antisera, indicating that neurotrophins may mediate these effects, perhaps in concert with other signals.  Ref: 12843289 J Neurosci, 2003
